# Potential Health Risks Posed by Plant-Derived Cumulative Neurotoxic Bufadienolides in South Africa

**DOI:** 10.3390/molecules21030348

**Published:** 2016-03-16

**Authors:** Christo Botha

**Affiliations:** Department of Paraclinical Sciences, Faculty of Veterinary Science, University of Pretoria, Private Bag X05, Onderstepoort 0110, South Africa; christo.botha@up.ac.za; Tel.: +27-12-529-8023; Fax: +27-12-529-8304

**Keywords:** cardiac glycosides, cotyledonosis, Crassulaceae, cumulative bufadienolides, human, “krimpsiekte”, risk

## Abstract

Bufadienolide-type cardiac glycosides have a worldwide distribution and are mainly synthesized by plants, but there are also animal sources. In South Africa, members of three genera of the Crassulaceae (*Cotyledon*, *Tylecodon* and *Kalanchoe*) cause a unique chronic form of cardiac glycoside poisoning, predominantly in small stock. This paretic/paralytic condition is referred to as “krimpsiekte”, cotyledonosis or “nenta”. “Krimpsiekte” is a plant poisoning only reported from South Africa and is regarded as the most important plant poisoning of small stock in the semi-arid Little Karoo and southern fringes of the Great Karoo. The toxicosis is caused by cumulative bufadienolides which have neurotoxic properties. Four types of cumulative neurotoxic bufadienolides, namely cotyledoside, and the tyledosides, orbicusides and lanceotoxins, have been isolated. Based on the structure activity relationships and certain toxicokinetic parameters possible reasons for their accumulation are presented. Consumption of edible tissues from animals that have ingested these plants poses a potential risk to humans.

## 1. Introduction

**Figure 1:**
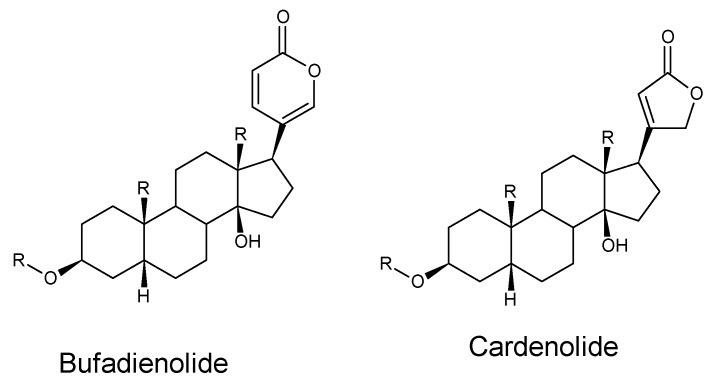
The basic chemical structures of the aglycones of bufadienolides and cardenolides.

Cardiac glycoside-containing plants have a worldwide distribution [1] (pp. 115–146). Chemically, two major groups of cardiac glycosides, namely the cardenolides and bufadienolides, are recognized (Figure 1) [2]. Bufadienolides are C-24 steroids and their glycosides and are characterized by a doubly unsaturated six-membered (pentadienolide) lactone ring located at C-17β [1,2,3]. However, the lactone ring of the cardenolides is a single unsaturated, five-membered (butenolide) ring at that position [1,2]. Numerous bufadienolides and their metabolites have been isolated, mainly from plants across the world, but also from toads of the genus *Bufo*, colubrid snakes (*Rhabdophis* species) and fireflies (*Photinus* species) [2,3,4]. Bufadienolide-containing plant species are members of the following families: Hyacinthaceae, Iridaceae, Melianthaceae, Ranunculaceae, Santalaceae, Crassulaceae, and Fabaceae [2,3]. There are comprehensive review articles available covering the chemistry of bufadienolides [2,3] and in addition to their use in traditional medicine, recent studies investigated the potential antineoplastic activities of a range of bufadienolides [3,4]. The interested reader is referred to these review articles.

In South Africa, poisoning of livestock by bufadienolide-containing plants has the greatest economic impact of all plant-associated poisonings. Various *Moraea* and *Drimia* species, regularly causes acute poisoning in livestock. On the other hand, a unique chronic form of bufadienolide poisoning is also recognized and is referred to as “krimpsiekte”, cotyledonosis, or “nenta” [1] (pp. 115–146). “Krimpsiekte” is an economically important syndrome, especially of sheep and goats, in the semi-arid, karoid regions of South Africa. This chronic neuromuscular affliction follows ingestion of certain members of the Crassulaceae. Only three genera of this family of succulents, *i.e.*, *Tylecodon*, *Cotyledon* and *Kalanchoe*, have thus far been confirmed to induce intoxication in small stock [5]. “Krimpsiekte” was one of the first veterinary diseases described in South Africa and was attributed to a poisonous plant when Veterinary Surgeon Jotello Soga reproduced the condition in 1891 by feeding *Tylecodon ventricosus* leaves to goats [6].

## 2. Plant Toxins

**Figure 2:**
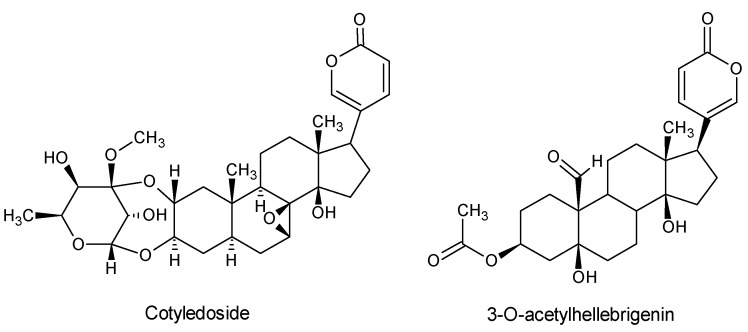

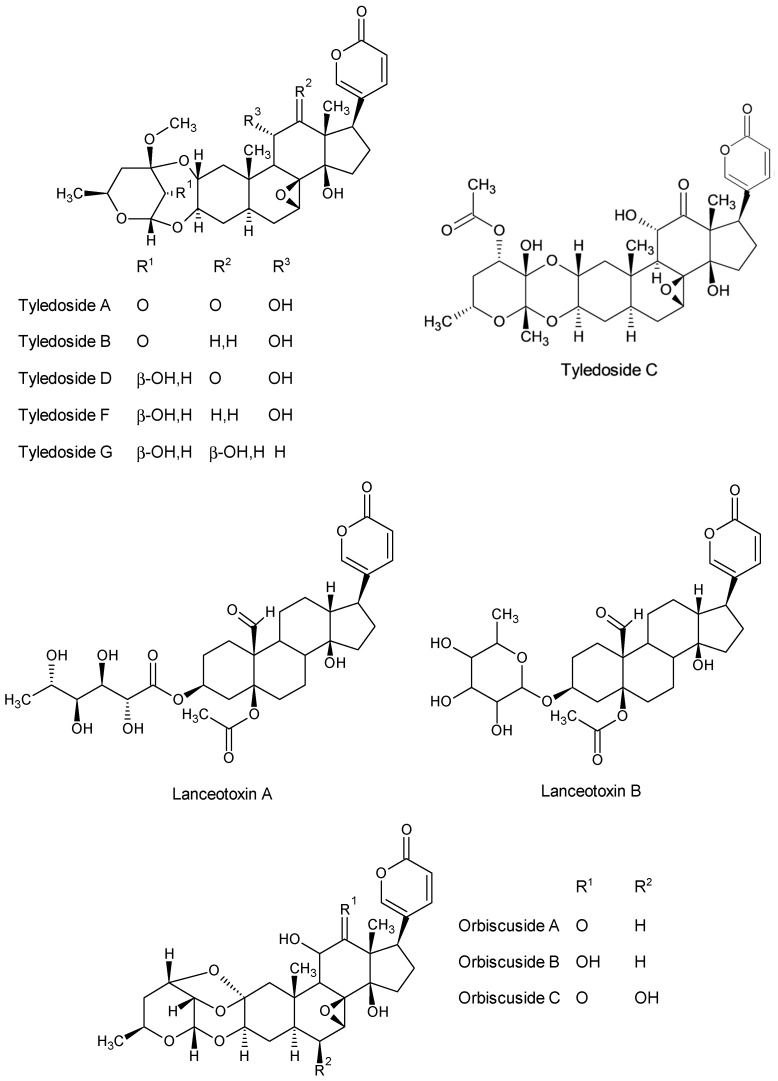
Bufadienolides isolated from poisonous members of the Crassulaceae in South Africa.

Various cumulative bufadienolides, with distinct neurotoxic properties (Figure 2), have been isolated from these succulents over the years [1] (pp. 115–146). The first bufadienolide-type of cardiac glycoside, namely cotyledoside, was isolated from *Tylecodon wallichii* [7,8]. Chronic intoxication in sheep (resembling field cases of “krimpsiekte”) was induced following two to five consecutive daily intravenous administrations of 0.01 mg cotyledoside/kg body weight [9]. The presence of cotyledoside in *T. wallichii* and the ability of this phytotoxin to induce chronic cardiac glycoside intoxication in sheep were later confirmed [10].

Four bufadienolides were isolated from *Cotyledon orbiculata*, namely tyledoside C as well as orbicusides A, B and C [11,12]. Five consecutive intravenous injections of 0.012 mg/kg orbicuside A induced “krimpsiekte” in a sheep [11].

Six bufadienolides isolated from *T. grandiflorus* were characterized as tyledosides A, B, C, D, F and G [13,14] and tyledoside D was also isolated from *T. ventricosus* [15]. Typical signs of “krimpsiekte” in a sheep were induced by five and four daily intravenous injections of 0.012 mg/kg of tyledosides A and D, respectively [13].

The presence of cardiac glycosides in *Kalanchoe lanceolata* was confirmed by the extraction and isolation of three bufadienolides, namely 3-*O*-acetylhellebrigenin, and the other two (initially referred to as K 28 A and K 28 B) were designated lanceotoxin A and lanceotoxin B [16,17]. “Krimpsiekte” was reproduced experimentally by intravenous administration of 0.01 mg/kg lanceotoxin B repeated six times over a 10 day period and 0.02 mg/kg lanceotoxin A, repeated four times over eight days [16].

## 3. Possible Structure-Activity Relationships (SAR)

As the bufadienolide-type of cardiac glycosides does not usually demonstrate an accumulative effect, it is suggested that the stereochemistry might explain the difference between a cumulative and non-cumulative effect [18]. The salient chemical characteristics of all four types of cumulative neurotoxic bufadienolides isolated from the Crassulaceae, *i.e.*, cotyledoside from *Tylecodon wallichii*, the tyledosides from *T. grandiflorus* and *T. ventricosus*, the orbicusides from *Cotyledon orbiculata* and lanceotoxins from *Kalanchoe lanceolata* (Figure 1) are as follows:

1. The sugar and aglycone moieties from all these cumulative glycosides cannot be removed by acid hydrolysis, which implies that the sugar is strongly bonded to the aglycone. In addition, in the first three types, the sugar is attached at the customary C-3 position, with additional ether bonds between the sugar and the C-2 position of the A-ring of the aglycone.
2. In all four types, the cumulative bufadienolides have laevorotatory sugars at the C-3positions of the A-rings.
3. An unusual epoxy-group over the C-7,8 position of the B-ring of the aglycone is common to all, except the lanceotoxins [18].

## 4. Clinical Signs

In “krimpsiekte” the cardiac, respiratory and gastrointestinal signs, typical of acute poisoning, diminish and the neuromuscular signs increases. This syndrome is broadly defined as a paretic/paralytic condition in small stock. Initially affected stock is disinclined to move or lags behind the flock, with the head nodding or dangling loosely, when driven. They tire easily and frequently lie down. The animals often assume a characteristic stance, with the feet together, the back arched and the head down, sometimes trembling. The Afrikaans term “krimpsiekte” actually refers to the tucked-in posture. The muscles of the neck are affected, often giving rise to torticollis, one of the notable diagnostic features. Frequent mouthing movements, drooping of the lower jaw, protrusion of the tongue, salivation and failure to masticate and swallow (resulting in half-chewed balls of ingesta in the mouth) complete the clinical picture. The animals are usually in poor condition ([1], (pp. 115–146), [5,10]).

## 5. Toxicokinetics

In a cotyledoside toxicokinetic study in sheep, a short plasma half-life was calculated and the cotyledoside plasma concentrations did not increase sequentially after five daily intravenous injections of 0.135 mg cotyledoside/kg body weight, nevertheless, the animals developed “krimpsiekte”. Thus, the anticipated cumulative effect of cotyledoside was not corroborated by increased plasma concentrations and it was concluded that there is a rapid distribution of cotyledoside to the extracellular fluid and/or tissues and a possible accumulation at the site of action [19].

## 6. Possible Mechanism of Action

The syndrome somewhat resembles myasthenia gravis as after light exercise the stock fatigues rapidly and frequently lies down [20]. It was proposed that the neuromuscular dysfunction could result from binding of these cumulative bufadienolides to nicotinic acetylcholine receptors at the neuromuscular junction [21]. A subsequent study indicated that the neurotoxic bufadienolides, cotyledoside and tyledoside D, are agonists at the muscarinic acetylcholine receptors of isolated rat jejunum. Based on this cholinergic activity it is postulated that during intoxication several neuromuscular receptors might be occupied by the cumulative bufadienolides resulting in a decreased number of functional nicotinic receptors. With continuous discharge at the motor nerve terminal acetylcholine stores are depleted. The few remaining unoccupied nicotinic receptors are soon occupied with acetylcholine and become desensitized to further stimulation. As a result of these two events fatigue could be expected to set in [21].

## 7. Potential Risk to Humans

The first report of secondary (relay) intoxication after ingestion of edible tissues from affected stock dates back as far as 1884, when neuromuscular signs were observed in a dog after feeding it on the livers of goats that died of “krimpsiekte” [22]. Later secondary poisoning was confirmed in dogs after they were fed goat and horse livers and horse meat obtained from “krimpsiekte” carcasses [23]. The toxic principle in edible tissue appears to be thermostable, not being destroyed at 120 °C for 15 min or by boiling in water for 30 min [23]. Natural cases of secondary poisoning have been reported in dogs when they fed on the viscera and meat of animals that have died of “krimpsiekte” [24] (pp. 720–734). This is of particular concern as secondary poisoning of humans can occur. Henning (1932) reported that the indigenous Bushmen and Hottentot people suffered from the disease following ingestion of “diseased meat” [24] (pp. 720–734). It is a real possibility because rural people in South Africa with meagre means and financial constraints do utilize the carcases of animals that have died and this is of grave concern. This aspect of “krimpsiekte” has not been properly investigated, despite its implications for public health.

The development of a prophylactic “krimpsiekte” vaccine has also received some attention [25]. One possible downside of inoculating small stock to prevent “krimpsiekte” is that due to the antibody-antigen binding, animals with high antibody titres may be able to accumulate more of these cumulative bufadienolides. When immunized animals that have ingested these plants are slaughtered, the edible tissues may contain residues and might, thus, not be fit for animal and human consumption [25].

There is a distinct possibility that these cumulative, neurotoxic bufadienolides might also be contained by other plant species around the world and scientists should be aware of the potential health risk posed by them. In general toxicologists should be aware of the potential for residues of natural toxins synthesized by poisonous plants, or even toxic metabolites of those natural toxins, to be present in edible tissues. Increased efforts should be made to calculate maximum residue levels and acceptable daily intakes of these natural toxins to safeguard consumers.

## References

[1] Kellerman T.S., Coetzer J.A.W., Naudé T.W., Botha C.J. (2005). Plant Poisonings and Mycotoxicoses of Livestock in Southern Africa.

[2] Steyn P.S., van Heerden F.R. (1998). Bufadienolides of plant and animal origin. Nat. Prod. Rep..

[3] Gao H., Popescu R., Kopp B., Wang Z. (2011). Bufadienolides and their antitumor activity. Nat. Prod. Rep..

[4] Kamboj A., Rathour A., Kaur M. (2013). Bufadienolides and their medicinal utility: A review. Int. J. Pharm. Pharm. Sci..

[5] Botha C.J. (2003). Krimpsiekte, a Paretic/Paralytic Syndrome of Small Stock, in South Africa: Studies on the Aetiology of Krimpsiekte, the Toxicokinetics and Dynamic effects of Cotyledoside and Seasonal Fluctuation of Cotyledoside Concentration in Plant Material. Ph.D. Thesis.

[6] Soga J.F. (1891). Disease “Nenta” in goats. Agric. J. Cape Good Hope.

[7] Van Rooyen G.F., Pieterse M.J. (1968). Die chemie van *Cotyledon wallichii* Harv. (kandelaarbos) II. Die isolering van’n bufadiënolied. J. S. Afr. Chem. I.

[8] Van Wyk A.J. (1975). The chemistry of *Cotyledon wallichii* Harv. Part III. The partial constitution of cotyledoside, a novel bufadienolide. J. S. Afr. Chem. I.

[9] Naudé T.W., Schultz R.A. (1982). Studies on South African cardiac glycosides II. Observations on the clinical and haemodynamic effects of cotyledoside. Onderstepoort J. Vet..

[10] Botha C.J., van der Lugt J.J., Erasmus G.L., Kellerman T.S., Schultz R.A., Vleggaar R. (1997). Krimpsiekte, associated with thalamic lesions, induced by the neurotoxic cardiac glycoside, cotyledoside, isolated from *Tylecodon wallichii* (Harv.) Toelken subsp. *wallichii*. Onderstepoort J. Vet..

[11] Anderson L.A.P., Schultz R.A., Kellerman T.S., Kotzé S.M., Prozesky L., Erasmus G.L., Labuschagne L. (1985). Isolation and characterization of and some observations on poisoning by bufadienolides from *Cotyledon orbiculata* L. var. *orbiculata*. Onderstepoort J. Vet..

[12] Steyn P.S., van Heerden F.R., Vleggaar R., Anderson L.A.P. (1986). Bufadienolide glycosides of the Crassulaceae. Structure and stereochemistry of orbicusides A–C, novel toxic metabolites of Cotyledon orbiculata. J. Chem. Soc. Perk. Trans. I.

[13] Anderson L.A.P., Joubert J.P.J., Prozesky L., Kellerman T.S., Schultz R.A., Procos J., Olivier P.M. (1983). The experimental production of krimpsiekte in sheep with *Tylecodon grandiflorus* (Burm. f.) Toelken and some of its bufadienolides. Onderstepoort J. Vet..

[14] Steyn P.S., van Heerden F.R., Vleggaar R., Anderson L.A.P. (1986). Structure elucidation and absolute configuration of the tyledosides, bufadienolide glycosides from *Tylecodon grandiflorus*. J. Chem. Soc. Perk. Trans. I.

[15] Botha C.J., Kellerman T.S., Schultz R.A., Erasmus G.L., Vleggaar R., Retief E. (1998). Krimpsiekte in a sheep following a single dose of *Tylecodon ventricosus* (Burm. f.) Toelken and the isolation of tyledoside D from this plant species. Onderstepoort J. Vet..

[16] Anderson L.A.P., Schultz R.A., Joubert J.P.J., Prozesky L., Kellerman T.S., Erasmus G.L., Procos J. (1983). Krimpsiekte and acute cardiac glycoside poisoning in sheep caused by bufadienolides from the plant *Kalanchoe lanceolata* Forssk. Onderstepoort J. Vet..

[17] Anderson L.A.P., Steyn P.S., van Heerden F.R. (1984). The characterization of two novel bufadienolides, lanceotoxins A and B from *Kalanchoe lanceolata* [Forssk.] Pers. J. Chem. Soc. Perk. Trans. I.

[18] Naudé T.W., Anderson L.A.P., Schultz R.A., Kellerman T.S., James L.F., Keeler R.F., Bailey E.M., Cheeke P.P., Hegarty M.P. (1992). Krimpsiekte: A chronic paralytic syndrome of small stock caused by cumulative bufadienolide cardiac glycosides. Poisonous Plants.

[19] Botha C.J., Rundberget T., Swan G.E., Mülders M.S.G., Flåøyen A. (2003). Toxicokinetics of cotyledoside following intravenous administration to sheep. J. S. Afr. Vet. Assoc..

[20] Gilhus N.E., Owe J.F., Hoff J.M., Romi F., Skeie G.O., Aarli J.A. (2011). Myasthenia gravis: A review of available treatment approaches. Autoimmune Dis..

[21] Botha C.J., Gehring R., van Rooyen J.M., Venter D. (2002). The effect of three bufadienolide cardiac glycosides on contraction of isolated rat jejenum. Onderstepoort J. Vet..

[22] Hutcheon D. (1899). Nenta. Agric. J. Cape Good Hope.

[23] Henning M.W. (1926). Krimpsiekte. 11th and 12th Reports of the Director of Veterinary Education and Research Part I.

[24] Henning M.W. (1932). Animal Diseases in South Africa.

[25] Botha C.J., Crafford J.E., Butler V.P., Stojanovic M.N., Labuschagne L. (2007). A potential krimpsiekte vaccine. Onderstepoort J. Vet..

